# Supplementary material for: FastqPuri: high-performance preprocessing of RNA-seq data
Source: BMC Bioinformatics. 2019 May 3;20:226. doi: 10.1186/s12859-019-2799-0 (PMC6500068; doi:10.1186/s12859-019-2799-0)
Supplement: Supplementary file 2 — Archive of FastqPuri. Archive containing all files needed to install and run FastqPuri v1.0.6. Date stamp March 22, 2019. (GZ 47,819 kb) [file 12859_2019_2799_MOESM2_ESM.gz › FastqPuri-1.0.6/html/init__makeTree_8h.html]

FastqPuri: include/init\_makeTree.h File Reference


FastqPuri


- include

Classes |
Typedefs |
Functions

init\_makeTree.h File Reference

Help dialog for makeTree and initialization of the command line arguments.
More...

`#include "defines.h"`

Include dependency graph for init\_makeTree.h:

This graph shows which files directly or indirectly include this file:

Go to the source code of this file.

|  |  |
| --- | --- |
| Classes | |
| struct | \_iparam\_makeTree |
|  | contains makeTree input parameters More... |
|  | |

|  |  |
| --- | --- |
| Typedefs | |
| typedef struct \_iparam\_makeTree | Iparam\_makeTree |
|  | contains makeTree input parameters |
|  | |

|  |  |
| --- | --- |
| Functions | |
| void | printHelpDialog\_makeTree () |
|  | Function that prints makeTree help dialog when called. |
|  | |
| void | getarg\_makeTree (int argc, char \*\*argv) |
|  | Reads in the arguments passed through the command line to makeTree. and stores them in the global variable par\_MT. |
|  | |

## Detailed Description

Help dialog for makeTree and initialization of the command line arguments.

Author
:   Paula Perez paula.nosp@m.pere.nosp@m.zrubi.nosp@m.o@gm.nosp@m.ail.c.nosp@m.om

Date
:   23.08.2017


---

Generated on Mon Mar 19 2018 23:42:01 for FastqPuri by  

 1.8.14
